# Can anemia predict perinatal outcomes in different stages of pregnancy?

**DOI:** 10.12669/pjms.326.11199

**Published:** 2016

**Authors:** Tayfun Vural, Emrah Toz, Aykut Ozcan, Alper Biler, Alper Ileri, Abdurrahman Hamdi Inan

**Affiliations:** 1 Tayfun Vural, Department of Obstetrics and Gynecology, Izmir Tepecik Education and Research Hospital, Izmir, Turkey; 2 Emrah Toz, Department of Obstetrics and Gynecology, Izmir Tepecik Education and Research Hospital, Izmir, Turkey; 3 Aykut Ozcan, Department of Obstetrics and Gynecology, Izmir Tepecik Education and Research Hospital, Izmir, Turkey; 4 Alper Biler, Department of Obstetrics and Gynecology, Izmir Tepecik Education and Research Hospital, Izmir, Turkey; 5 Alper Ileri, Department of Obstetrics and Gynecology, Izmir Buca Maternity and Children Hospital, Izmir, Turkey; 6 Abdurrahman Hamdi Inan, Department of Obstetrics and Gynecology, Izmir Tepecik Education and Research Hospital, Izmir, Turkey

**Keywords:** Anemia, Preterm delivery, Low birth weight

## Abstract

**Objective:**

To investigate the effect of anemia on perinatal outcomes as preterm delivery (PTD) and low birth weight (LBW) in the different stages of pregnancy.

**Methods:**

Medical records of 39,587 Turkish pregnant women who delivered between January 2011 and September 2014 were reviewed. Anemia during pregnancy was defined as hemoglobin (Hb)< 11 g/dl, low birth weight was defined as birth weight <2500 gr and PTD was defined as <37 weeks. The pregnant women were divided into three groups (Hb<10 gr/dl, Hb 10-11 gr/dl, Hb>11 gr/dl). Perinatal outcomes were compared between these anemic and non-anemic groups.

**Results:**

The anemia prevalence in our study was 25.1%. In the first and second trimester of Hb<10g/dl group LBW ratio was significantly higher (respectively 13.5%, 9.8%, p=0.03; 22.7%, 14.7%, p=0.01). In the second and third trimesters of Hb<10 g/dl group PTD ratio was significantly higher (respectively 29.1%, 19%, p=0.00; 17.7%, 15.4% p=0.02). In the first trimester Hb<10 g/dl group cesarean section rate was significantly higher (respectively 65.2%, 57.6%, p = 0.01).

**Conclusions:**

LBW infants and preterm birth rate was higher in Hb <10 gr/dl group than non-anemic in the first, second and third trimester. Hb <10 gr/dl group had higher cesarean rate in first trimester. The mean birth weight was significantly lower in anemic pregnant women in the second trimester. Preterm birth and cesarean section rate, in the group of anemic throughout pregnancy was higher than those of non-anemic in the whole pregnancy period.

## INTRODUCTION

Anemia is seen in different populations, with different etiology, incidence and severity and the most common medical disorder in pregnancy.^1^ Half of pregnant women are anemic worldwide.^2^ Several studies have reported that maternal anemia during pregnancy is a risk factor for adverse perinatal outcomes such as preterm birth, low birth weight (LBW).^3^-^5^ While some reports found significant association between adverse perinatal outcomes and anemia others have refuted the claim.^6^,^7^ This has led to a questioning of iron supplementation in pregnancy whether necessary.^8^,^9^

Different results in the studies depend on variables such as the study design, different diagnostic criterias, demographic characteristics in different societies. In many societies anemic women have other features (such as low socio-economic status) which may cause low birth weight and preterm birth. Therefore, as noted in several studies in the literature, it is difficult to eliminate these differences between anemic and non-anemic pregnant women and design an independent study.^4^,^5^ Hemoglobin declines due to physiologic expansion of the plasma volume during pregnancy.^10^,^11^ Accordingly, hemoglobin values were defined as <11 g/dl in pregnancy while the limit was <12 gr/dl for anemia in non-pregnant women.^12^

The aim of this study was to investigate the impact of anemia on pregnancy outcomes in different stages of pregnancy. Correlation between anemia and pregnancy outcomes in pregnancy may provide guidance as to whether is required iron supplementation.

## METHODS

This retrospective study covers 39,587 women performed follow-ups during pregnancy and gave birth in our hospital between January 2011 and September 2014. Individuals who didn’t come to regular antenatal care, had multiple pregnancies or congenital malformations, and pregnant women with systemic diseases (diabetes, preeclampsia / eclampsia, endocrinopathy, systemic lupus erythematosus, hemoglobinopathies) were excluded from the study.

Hemoglobin levels of first, second and third trimester and Hb levels of the patients admitted to the delivery room for birth were obtained from a tertiary hospital centers with our computerized medical records system. The women were divided into three groups according to their hemoglobin levels (Hb < 10gr/dl, Hb 10-11 gr/dl, Hb > 11gr/dl). Hb values are measured by Coulter® LH 780 Hematology Analyzer (Beckman Coulter, Miami, United States) and cyanmethaemoglobin method in our hospital.

In our study infants born <2500 g was considered as LBW and <37 weeks of gestation was considered as preterm birth. Iron supplementation was prescribed regularly to all pregnant women in our hospital. 30 mg/day of elemental iron to non-anemics while 30-120mg/day of elemental iron to anemics was provided. The study has been conducted in accordance with the Declaration of Helsinki.

In this study to compare categorical (qualitative) variables, Fisher’s exact test was used, where appropriate. For the comparison of quantitative (continuous) variables, t-test was used. The percentage difference between groups is calculated with Chi-square (χ^2^) test for statistical significance. All analyzes were performed using SPSS 20.0 statistical package.

## RESULTS

In this retrospective study 39 587 participants and their newborns were evaluated. Mean maternal age: 27.56 ± 6.05 SD, mean gestational age at delivery: 38.24± 2.88 SD, mean birth weight was 3.133 ± 683 gr SD, mean Hb: 11.59 gr/dl, maximum Hb: 16.4 gr/dl, minimum Hb:5.9 gr/dl, the prevalence of anemia was % 25.12 in our study. The demographic characteristics of our study group are summarized in Table-I.

**Table-I:** Demographic characteristics of patients.

		Hb ≥ 11 gr/dL		Hb < 11 gr/dL		Total		p
Maternal Age		27.69±6.04		27.18±6.08		27.56±6.05		0.01*
Parity	1	12968	43.1%	4329	43.0%	17297	43.1%	0.987
	2-4	16155	53.7%	5417	53.8%	21572	53.7%	
	5+	728	2.4%	246	2.4%	974	2.4%	
Mode of Delivery	Normal Vaginal	13631	46.0%	4213	42.2%	17844	45.1%	0.01*
	Cesarean	15974	54.0%	5769	57.8%	21743	54.9%	
Number of curettage	0	3901	63.9%	1116	59.5%	5017	62.9%	0.01*
	1	1685	27.6%	612	32.6%	2297	28.8%	
	2+	516	8.5%	147	7.8%	663	8.3%	
Number of abortion	0	2989	36.4%	912	34.1%	3901	35.8%	0.085*
	1	3935	47.9%	1337	49.9%	5272	48.4%	
	2+	1285	15.7%	428	16.0%	1713	15.7%	

Hb: Hemoglobin

* p-values < 0,05 were considered statistically significant.

Prevalence of anemia in the patients who gave birth in first, second and third trimesters were 11.9%, 32.1%, 33% respectively. The prevalence of anemia increased while pregnancy proceed. Prevalence of maternal anaemia was 26.1% at delivery.

Cesarean rate was 65.2% in the group with Hb <10 gr/dl in the first trimester and significantly higher than in non-anemic group (57.6%) (p:0.01). LBW infants ratio was 13.5% in the same group and also considerably higher than in non-anemic women (%9.8) (p:0.03). The mean birth weight was 3120 grams in the anemic group. In the non-anemic group it was 3187 grams (p:0.06). p-values =0,1 were considered statistically significant. Particularly we can state that although it is very close to cut off value, birth weight in anemic group was lower than in non-anemic group (Table-II).

**Table-II:** Comparison of anemic and non-anemic patients hemoglobin values and perinatal outcomes.

	Mode of Delivery				LBW				Preterm				Birth Weight	

	Normal Vaginal		Cesarean		No		Yes		No		Yes			

	N	%	N	%	N	%	N	%	N	%	N	%	Mean	SDS
Anemia 1.Tr														
Hb < 10 gr/dl	113	2.8%	212	3.8%	283	3.3%	44	4.6%	274	3.3%	53	3.9%	3120.34	739.66
Hb 10-11 gr/dl	348	8.7%	470	8.5%	741	8.5%	83	8.6%	696	8.4%	128	9.5%	3150.51	686.23
Hb > 11 gr/dl	3555	88.5%	4828	87.6%	7673	88.2%	838	86.8%	7342	88.3%	1169	86.6%	3187.43	627.56
p	0.02				0.102				0.18				0.01	
Anemia 2.Tr														
Hb < 10 gr/dl	441	9.5%	597	9.4%	812	8.7%	238	13.3%	744	8.4%	306	13.1%	2862.23	980.99
Hb 10-11 gr/dl	1084	23.4%	1414	22.3%	2088	22.3%	439	24.5%	1940	22.0%	587	25.2%	3002.66	876.70
Hb > 11 gr/dl	3114	67.1%	4323	68.3%	6448	69.0%	1112	62.2%	6121	69.5%	1439	61.7%	3068.39	810.47
p	0.41				0.01				0.01				0.05	
Anemia 3.Tr														
Hb < 10 gr/dl	1187	12.3%	1714	12.5%	2578	12.3%	371	13.7%	2427	12.1%	522	13.9%	3163.81	627.99
Hb 10-11 gr/dl	2009	20.8%	2820	20.5%	4392	20.9%	509	18.8%	4110	20.6%	791	21.0%	3189.45	596.48
Hb > 11 gr/dl	6479	67.0%	9199	67.0%	14073	66.9%	1829	67.5%	13452	67.3%	2450	65.1%	3173.70	624.17
p	0.84				0.01				0.01				0.04	

Tr: Trimester, Hb: Hemoglobin, LBW: Low Birth Weight, SDS: Standart Deviation Scores.

LBW rates in the second trimester were 22.7% and 17.4% in the groups of Hb <10 gr/dl, Hb 10-11 gr/dl, respectively, LBW (14.7%) was significantly higher in non-anemic women (p<0.01). In second trimester groups of Hb <10 gr/dl, Hb 10-11 gr/dl the preterm birth rates were 29.1%, 23.2%, respectively and according to the preterm birth rate they were significantly higher compared to non-anemic group (19%) (p<0.01). Average birth weights were 2862 gr, 3002 gr in the Hb <10 gr/dl, Hb 10-11 gr/dl groups, respectively, they were notably lower compared to non-anemic mothers (3068 gr) (p< 0.01vs p: 0.005). The average birth weight increased while Hb values increased.

In the third trimester Hb <10 gr/dl group’s LBW infant rate was 12.6%, also significantly higher than in non-anemic women (11.5%)(p:0.1). Hb <10 gr/dl group preterm birth rate was 17.7% and considerably higher when compared non-anemic group (15.4%) (p:0.02).

Comparing the trimesters of anemic pregnant women, in the first trimester Hb <10 gr/dl group cesarean rate was 65.2%, significantly higher than in 2nd and 3rd trimester caesarean section rate (58.1%) which carried same hemoglobin value (p<0.01) (Table-III). Hb <10 gr/dl in the first trimester was determined as risk factor for cesarean section. Prevention of anemia in the first trimester is crucial for reducing the cesarean rate.

**Table-III:** Comparison of anemic patients in terms of delivery mode and perinatal outcomes.

	1.Tr Hb<10		2&3 Tr Hb < 10		1.Tr Hb (10-11)		2&3 Tr Hb (10-11)		(During Pregnancy) Hb < 11 gr/dL		(During Pregnancy) Hb > 11 gr/dL	

	N	%	N	%	N	%	N	%	N	%	N	%
Mode of Delivery												
Normal Vaginal	113	34.8%	1230	41.9%	348	42.5%	2521	42.7%	1879	37.6%	9349	57.0%
Cesarean	212	65.2%	1703	58.1%	470	57.5%	3384	57.3%	3123	62.4%	7058	43.0%
p	0.01				0.92				0.001			
LBW												
No	283	86.5%	2504	84.0%	741	89.9%	5197	86.6%	4395	87.1%	14739	87.8%
Yes	44	13.5%	477	16.0%	83	10.1%	801	13.4%	652	12.9%	2040	12.2%
p	0.24				0.01				0.187			
Preterm												
No	274	83.8%	2344	78.6%	696	84.5%	4871	81.2%	4085	80.9%	14186	84.5%
Yes	53	16.2%	637	21.4%	128	15.5%	1127	18.8%	962	19.1%	2593	15.5%
p	0.03				0.03				0.001			
												
Weight	N	mean±SDS	N	mean±SDS	N	mean±SDS	N	mean±SDS	N	mean±SDS	N	mean±SDS
	327	3120±740	2981	3069±767	824	3150±686	5998	3112±726	5047	3142±639	16779	3139±643
P	0.25				0.15				0.770			

Hb: Hemoglobin, Tr: Trimester, LBW: Low Birth Weight, SDS: Standart Deviation Scores.

In second and third trimester preterm birth rates were 21.4%, 18.8% in Hb <10 gr/dl, Hb 10-11 gr/dl groups, respectively, they were significantly higher than the group which carried the same hemoglobin levels in the first semester (16.2%, 15.4% respectively) (p:0.03). 2nd and 3rd trimester anemia was more dangerous than in the first trimester for preterm birth (Table-III).

The anemic mothers’, throughout the pregnancy from the early stage of pregnancy (first trimester) until the end of pregnancy (end of the third trimester), cesarean rate was (62.4%) significantly higher than in the non-anemic group (43%) (p<0.01). Preterm birth rate in anemic mothers during pregnancy was 19.1%, considerably higher than the rate of preterm birth in non-anemic group (15.5%) (p<0.01) (Table-III).

## DISCUSSION

The World Health Organization (WHO) defined the hemoglobin levels of anemia in pregnancy as <11 gr/dl in 2001.12 In our study, the prevalence of anemia in pregnancy has been reported as 25%.12 The estimated prevalence of anaemia in pregnancy differs widely, 58% in China, 50% in South Asia, 40% in Istanbul.^13^-^15^ Anemia is seen at different frequencies in different populations. In our study reasons for the lower prevalence of anemia than the literature can be listed as iron supplementation was routinely prescribed to all pregnant women in our hospital and majority of the study group patients were regular prenatal clinic attendees.

Traditionally, anemia is associated with suboptimal pregnancy outcomes such as low birth weight and preterm birth.^4^,^5^ First, second and third trimester group of Hb <10 gr/dl; LBW infants, preterm birth rate is higher than non-anemic women and mean birth weight is lower. Also in the group with anemic throughout the entire pregnancy, cesarean section and preterm birth rate is higher than non-anemic. In our study, there is a strong association between anemia with preterm birth and LBW infant.

Anemia during pregnancy is more common in patients with high parity. In literature, the importance of the multi birth and the frequent birth has been reported in the etiology of anemia.^16^-^18^ Anemia develops when increased iron needs couldn’t be met during pregnancy.^16^,^19^ Increasing plasma volume more than red blood cell mass causes physiologic fall in the hematocrit.^16^ Iron which is stemming from poor iron reserve of pregnant women with inadequate dietary intake of iron, does not meet the growing need. Iron reserves are depleted due to recurrent pregnancies. Anemia effect gradually increases due to growing fetus iron need compensation from the mother directly.^20^

What is interesting in the case of pregnancy is, high hemoglobin levels are not always with positive perinatal outcomes. There is U shape relationship between maternal hemoglobin levels and perinatal outcomes. Thus, both high and low hemoglobin levels are associated with adverse perinatal outcomes.^21^

Despite the increase in the total number of erythrocytes, hemoglobin concentration decreased because of plasma volume during pregnancy were estimated to increase by more than the total erythrocyte mass. So placental perfusion become more favorable for maternal -fetal gas and nutrient exchange with reduced blood viscosity.^22^

Increased viscosity secondary to high maternal hemoglobin levels can create placental infarcts. Large placental infarction has been reported to lead to intrauterine growth retardation and perinatal death.^23^ Breymann’s study reported increase risk for the preeclampsia and SGA (small for gestational age) is associated with low plasma volume with Hb> 12 gr/dl levels in the late second trimester. In the same study ideal hemoglobin levels to prevent preterm birth and SGA are considered as 9.5-11.5 gr/dl.^24^

Critical point in the iron treatment is to assess the actual iron requirement for each pregnancy accurately. Because iron results in production of free radicals and placental oxidative stress.^25^

### Limitations of this study

Difficulty in planning independent study by eliminating differences with additional features that may lead to negative perinatal outcomes (low socio-economic status, etc.) in most anemic pregnant; Not questioning the usage although given routine iron supplementation to all pregnant women as hospital policy; Not investigating the relationship between birth weights and physical properties of parents. The strengths of our study is that it is quite adequate and homogeneous in terms of demographic statistical inferences number of patients. Moreover, several studies in the literature have investigated the effects of anemia in pregnancy by certain period, but in our study perinatal effects of anemia was investigated in all three trimesters of pregnancy.

## CONCLUSIONS

LBW infants and preterm birth rate was higher in Hb <10 gr/dl group than non-anemic in the first, second and third trimester. Hb <10 gr/dl group had higher cesarean rate in first trimester. The mean birth weight was significantly lower in anemic pregnant women in the second trimester. Hb <10 gr/dl group’s preterm birth rate was higher in 2nd and 3rd trimester than the group with the same hemoglobin levels in the first trimester. Namely, with <10 gr/dl hemoglobin levels, preterm delivery risk was higher in 2nd and 3rd trimester than in the first trimester. Preterm birth and cesarean section rate, in the group of anemic throughout pregnancy was higher than those of non-anemic in the whole pregnancy period.
